# Multidimensional Separation Using HILIC and SCX Pre-fractionation for RP LC-MS/MS Platform with Automated Exclusion List-based MS Data Acquisition with Increased Protein Quantification

**DOI:** 10.4172/jpb.1000378

**Published:** 2015-11-28

**Authors:** Yu Zhou, Zhen Meng, Maria Edman-Woolcott, Sarah F Hamm-Alvarez, Ebrahim Zandi

**Affiliations:** 1 USC Research Center for Liver Diseases, Keck School of Medicine, University of Southern California, Los Angeles, California, USA; 2 Norris Comprehensive Cancer Center, Department of Molecular Microbiology and Immunology, Keck School of Medicine, University of Southern California, Los Angeles, California, USA; 3 Department of Ophthalmology, Keck School of Medicine, University of Southern California, Los Angeles, California, USA; 4 Department of Pharmacology and Pharmaceutical Sciences, School of Pharmacy, University of Southern California, 1985 Zonal Ave, Los Angeles, California, USA

**Keywords:** Proteomics, Biomarkers, TMT quantification, Exclusion list-based MS data acquisition, HILIC, SCX

## Abstract

Liquid chromatography–mass spectrometry (LC-MS) based proteomics is one of the most widely used analytical platforms for global protein discovery and quantification. One of the challenges is the difficulty of identifying low abundance biomarker proteins from limited biological samples. Extensive fractionation could expand proteomics dynamic range, however, at the cost of high sample and time consumption. Extensive fractionation would increase the sample need and the labeling cost. Also quantitative proteomics depending on high resolution MS have the limitation of spectral acquisition speed. Those practical problems hinder the in-depth quantitative proteomics analysis such as tandem mass tag (TMT) experiments. We found the joint use of hydrophilic interaction liquid chromatography (HILIC) and strong cation exchange Chromatography (SCX) prefractionation at medium level could improve MS/MS efficiency, increase proteome coverage, shorten analysis time and save valuable samples. In addition, we scripted a program, Exclusion List Convertor (ELC), which automates and streamlines data acquisition workflow using the precursor ion exclusion (PIE) method. PIE reduces redundancy of high abundance MS/MS analyses by running replicates of the sample. The precursor ions detected in the initial run(s) are excluded for MS/MS in the subsequent run. We compared PIE methods with standard data dependent acquisition (DDA) methods running replicates without PIE for their effectiveness in quantifying TMT-tagged peptides and proteins in mouse tears. We quantified a total of 845 proteins and 1401 peptides using the PIE workflow, while the DDA method only resulted in 347 proteins and 731 peptides. This represents a 144% increase of protein identifications as a result of PIE analysis.

## Introduction

Protein expression changes from animal models and humans can provide functional insight into pathological processes of disease and therapeutic responses, and therefore serve as useful biomarkers. Quantitative mass spectrometry-based proteomic profiling is one of the emerging technologies for protein biomarker discovery, quantification and analysis [1,2]. However, a full implementation of this technology to profile and quantify an entire proteome from biological samples is not possible yet due to technological limitations. There are still many challenges that hamper the true power of this technology for protein biomarker discovery and quantitative comparison of various samples with complex proteomes [3,4]. The dynamic concentration range of proteins in biological samples can reach eleven orders of magnitudes [5]. A comprehensive analysis of such complex proteomes far exceeds the current capabilities of mass spectrometry-based proteomics technologies. A widely used strategy to reduce the proteome complexity is extensive fractionation including various chromatography techniques, affinity purification, and immuno-depletion of samples prior to MS analysis [6,7]. These techniques can effectively reduce sample complexity, but they are also limited by availability of antibodies, small quantities of starting materials, and there is potential for sample loss [8].

Improving instrument properties such as ion injection efficiency, cycling speed and detector sensitivity has been suggested to increase the efficiency of proteomics analysis [9]. It has been shown that the current quantitative data acquisition platforms bias identification towards high-abundance proteins. It would often redundantly sample high-intensity precursor ions while failing to sample low-intensity precursors entirely. As many disease-relevant proteins, including signaling and regulatory proteins, are typically expressed at low levels, this tends to limit the acquisition of the most-valuable information. Even with dynamic exclusion and new instrumentation, LC-ESI MS still has intrinsic limitations when analyzing complex samples, as the number of peptide ions entering the mass analyzer significantly exceeds the available sequencing cycles of the mass spectrometer. For example, Orbitrap, the instrument of choice for TMT tandem mass tagging quantification, has a low scanning rate using CID/HCD dual at high/high mode [9]. Because of the extra time needed for HCD analysis, the duty cycle of MS2 acquisition is significantly lower in the CID-HCD dual-scan configuration than the CID-only configuration. Therefore, the potential for MS under-sampling is much greater when the analysis is performed at high/high mode for quantitative survey scan.

Thus limitations such as low amount of readily available samples, the need of extensive fractionation, and low MS scanning rate for quantitative data acquisition still present significant challenges for large-scale quantitative mass spectrometry-based proteomics.

To overcome some of these technological hurdles and advance quantitative capabilities, an improved Precursor Ion Exclusion List (PIE) MS data acquisition combined with a simplified HILIC and SCX fractionation is presented in this study. The aim of this study was to develop and evaluate a more efficient method to profile the tear proteome, and to maximize protein quantifications while minimizing redundancies in serial analyses.

Precursor ion exclusion (PIE) MS data acquisition is a useful concept and has been applied to different MS platforms [10–12]. Using PIE, masses of successfully identified peptides are used to generate an exclusion list such that those precursors are not selected for sequencing during subsequent analyses.

The Thermo PD exclusion list export function facilitates the utilization of the PIE method. However, without merging molecular weight and retention time cluster, this list cannot be imported directly into exclusion list table for the next iteration method. In practice, it may take several hours of manual processing of exclusion lists for the next individual PIE-based method for complex samples, which reduces the throughput and automation of workflow significantly. To address this practical problem, we scripted a program, Exclusion List Convertor (ELC), which automates and streamlines data acquisition workflow using the PIE method. ELC merges molecular weight and retention time cluster, and it is used to convert the PD exclusion list results to a format, which is suitable for import into the LTQ-Orbitrap XL data acquisition method for next PIE-based iteration without manual intervention. ELC also merges the exclusion list with the previous list and can be applied to the acquisition of the next LC-MS/MS run.

The PIE method necessitates sufficient sample quantities for iteration runs. However limited starting materials are a problem for extensive fractionation and multiple MS runs. To overcome this challenge, we used a simplified and complementary HILIC and SCX fractionation method at a medium level. This complementary fractionation approach provides not only increased resolving power of a complex proteome, but also sufficient fractionated sample quantities for multiple MS runs with PIE iterations. We hypothesized that integration of complementary HILIC /SCX fractionation with the PIE method would minimize redundant MS/MS acquisitions, thus facilitating analysis of low-intensity peptides from samples of limited quantity.

## Materials and Method

### Sample preparation

The tear proteins were first digested with Lys-C (Roche Diagnostics, Ingelheim, Germany) at an enzyme:protein ratio of 1:50 for 5 h at 37°C, and this was followed by 4× dilution of the samples with 50 mm triethyl ammonium bicarbonate and digestion with trypsin (Roche Diagnostics), at an enzyme:protein ratio of 1:100, overnight at 37°C. Peptides were labeled with TMTs using the TMT 6-plex labeling kit (Pierce) according to the manufacturer’s instructions.

### Offline SCX and HILIC fractionation

We used SCX and HILIC spin cartridges from Nestgroup according to manufactures instructions. Six fractions for each HILIC and SCX were collected. For SCX, we loaded samples using 25% ACN and 0.05% Formic Acid. Then we eluted the fractions using 6 salt steps of ammonium formate (50 mM, 100 mM, 150 mM, 200 mM, 300 mM, and 400 mM) in loading buffer. For HILIC, we loaded samples using 85% ACN and 15 mM ammonium formate at pH 6. We eluted the fractions using 6 steps of ACN (80%, 75%, 70%, 65%, 60%, and 40%) in 15 mM ammonium formate. After digestion a C18 clean-up using ziptip (Thermo) was performed on all samples according to the manufacturer’s instructions.

### LC/MS/MS analysis

Samples were analyzed using an LC/MS system consisting of an Eksigent NanoLC Ultra 2D (Dublin, CA) and Thermo Fisher Scientific LTQ Orbitrap XL (San Jose, CA). Briefly, peptides were separated in a 10 cm column (75 µm inner diameter) packed in-house with 5 µm C18 beads on a Eksigent NanoLC Ultra 2D system using a binary gradient of buffer A (0.1% formic acid) and buffer B (0.1% formic acid and 80% ACN). The peptides were loaded directly without any trapping column with buffer A at a flow rate of 300 nL/min. Elution was carried out at a flow rate of 250 nL/min, with a linear gradient from 10% to 35% buffer B in 95 min followed by 50% B for 15 min. At the end of the gradient, the column was washed with 90% B and equilibrated with 5% B for 10 min. The eluted peptides were sprayed into the LTQ Orbitrap XL.

The source was operated at 2.1–2.25 kV, with no sheath gas flow, with the ion transfer tube at 250°C. MS spectra in the range of m/z 350–2000 were acquired in the orbitrap at a FWHM resolution of 60,000 after accumulation to an AGC target value of 500,000 in the linear ion trap with 1 microscan.

The mass spectrometer was operated in the data-dependent acquisition mode for DDA experiment, and exclusion list-based data acquisition mode for PIE experiment. For all sequencing events, dynamic exclusion was enabled to minimize repeated sequencing. Peaks selected for fragmentation more than once within 60 s were excluded from selection (10 ppm window).

### Data processing and analysis

Proteome Discoverer 1.3 (Thermo Fisher Scientific) was used for protein identification using Sequest algorithms. The following criteria were followed. The variable modifications used were carbamidomethylation(C), oxidation (M), TMT 6-plex (K), and TMT 6-plex (N-term). Searches were conducted against Uniprot mouse or in-house customer database. Up to two missed cleavages were allowed for protease digestion and peptide had to be fully tryptic. MS1 tolerance was 10 ppm and MS2 tolerance was set at 0.8 Da. Peptides reported via search engine were accepted only if they met the false discovery rate of 1%. There is no fixed cutoff score threshold, but instead spectra are accepted until the 1% FDR rate is reached. Only peptides with a minimum of six amino acid lengths were considered for identification. We also validated the identifications by manual inspection of the mass spectra.

### Ingenuity pathway analysis to determine the proteins related to diseases and bio functions implicated in Hermansky-Pudlak syndrome mouse model

The diseases and bio functions analyses were generated through the use of QIAGEN’s Ingenuity Pathway Analysis (IPA^®^, QIAGEN Redwood City, www.qiagen.com/ingenuity).

The IPA knowledge base consists of a high-quality expert-curated database containing 1.5 million biological findings consisting of more than 42,000 mammalian genes and pathway interactions extracted from the literature. In brief, proteins that were confidently identified were considered for IPA analysis. Ingenuity then used these proteins and their identifiers to navigate the curated literature database and extract the biological relevant information among the candidate proteins. Associated diseases and bio functions were generated, along with a score representing the log probability of a particular disease and bio function being found by random chance. Top diseases and bio functions associated with the uploaded data were presented, along with a p value. The p values were calculated using right-tailed Fisher’s exact tests.

## Results and Discussion

**Figure 1:**
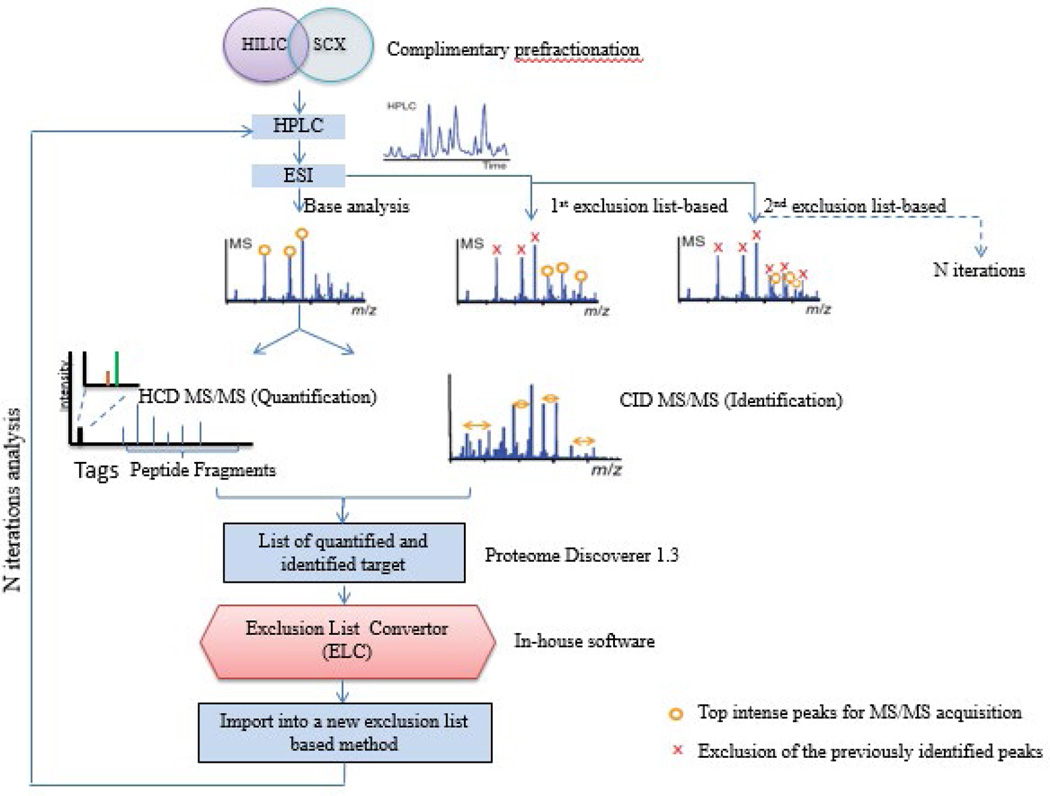
Workflow of a precursor ion exclusion-based proteomic experiment and streamlined TMT data acquisition using Exclusion List Convertor (in-house software). We used a top 3 × 2 method for LTQ-Orbitrap XL data acquisition, which consists of three HCD events followed by three CID events on the same ions selected for the first three HCD events from high resolution MS survey scan. Quantification is based on low *m/z* reporter fragment ion intensities from HCD MS/MS, and CID MS/MS enables the peptide sequence to be determined. Exclusion list-based method enables the analysis of same samples but with exclusion of the previously identified peptides. A C++ script (Exclusion List Convertor), which could merge molecular weight and retention time cluster, was used to convert PD 1.3 exclusion list results to a format which was suitable to import into the LTQ-Orbitrap XL data acquisition method for next exclusion list-based iteration without manual intervention. Exclusion List Convertor (ELC) also can merge the exclusion list with the previous list and applied to the acquisition of the next LC-MS/MS run.

We integrated complementary HILIC/SCX fractionation with the PIE method, which could minimize redundant MS/MS acquisitions, thus facilitate analysis of low-intensity peptides from samples of limited quantity (Figure 1).

We tested this strategy for quantitative proteome analysis of mouse tear fluid, which by nature is available in limited quantities. We used tear samples from pearl mouse (B6Pin.C3-Ap3b1pe/J, Jackson Laboratory, Bar Harbor, ME), with spontaneously occurring mutations in the β3A subunit gene (Ap3b1) of the AP-3 adaptor complex. These mice are models for Hermansky-Pudlak syndrome, whose symptoms include hypopigmentation, lysosomal secretion abnormalities and prolonged bleeding due to platelet storage pool deficiency.

**Figure 2:**
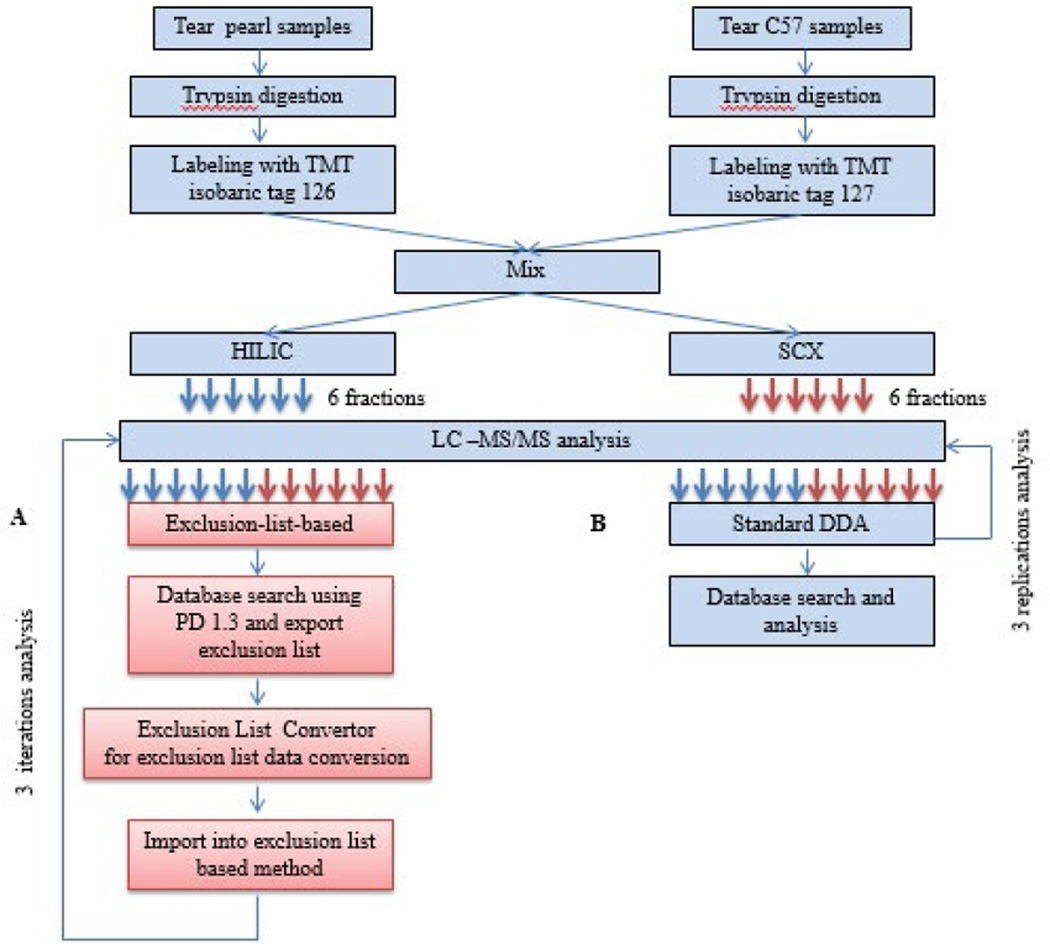
Schematic comparison of the evaluated Exclusion list-based and standard DDA strategies. First, tear samples from pearl or C57 mouse were digested into peptides using Lys-C and trypsin. The resulting peptides were acidified and then desalted using a Sep-Pak C18 column. After labeled with TMT isobaric tag, aliquots were mixed for HILIC and SCX pre-fractionation. (A) In the exclusion list based strategy, 6 fractions of HILIC and 6 fractions of SCX were subjected to a 2 h LC–MS analysis using in house software to simplify and streamline the creation of methods for subsequent iteration analysis. (B) In the standard DDA strategy, aliquots of the same samples as exclusion list-based strategy were subjected to 2 h LC–MS analyses with 3 replicates.

The schematic representation of the experimental design is shown in Figure 2. First, 100 µg tear samples from pearl or C57BL/6 (C57) mice were digested into peptides using Lys-C and trypsin. The resulting peptides were acidified and then desalted using a Sep-Pak C18 column. Then peptides were derivatized with isobaric TMT tandem mass tagging reagent (Thermo Fischer Scientific, Waltham, MA). In the duplex TMT experiments, tryptic peptides from pearl mouse were labeled with m/z 126 isobaric TMT tag, while those from C57 mice was labeled with m/z 127 isobaric TMT tag. The two labeled peptide pools were mixed in a 1:1 ratio.

We performed offline fractionation using complementary HILIC and SCX cartridges. We compared PIE method side by side with standard data dependent acquisition (DDA). Equal amount of each fraction was analyzed by both PIE-based workflow and DDA workflow. The instrument setups for LC MS/MS runs were identical for both DDA and PIE methods, with exception of dynamic exclusion setting.

**Figure 3:**
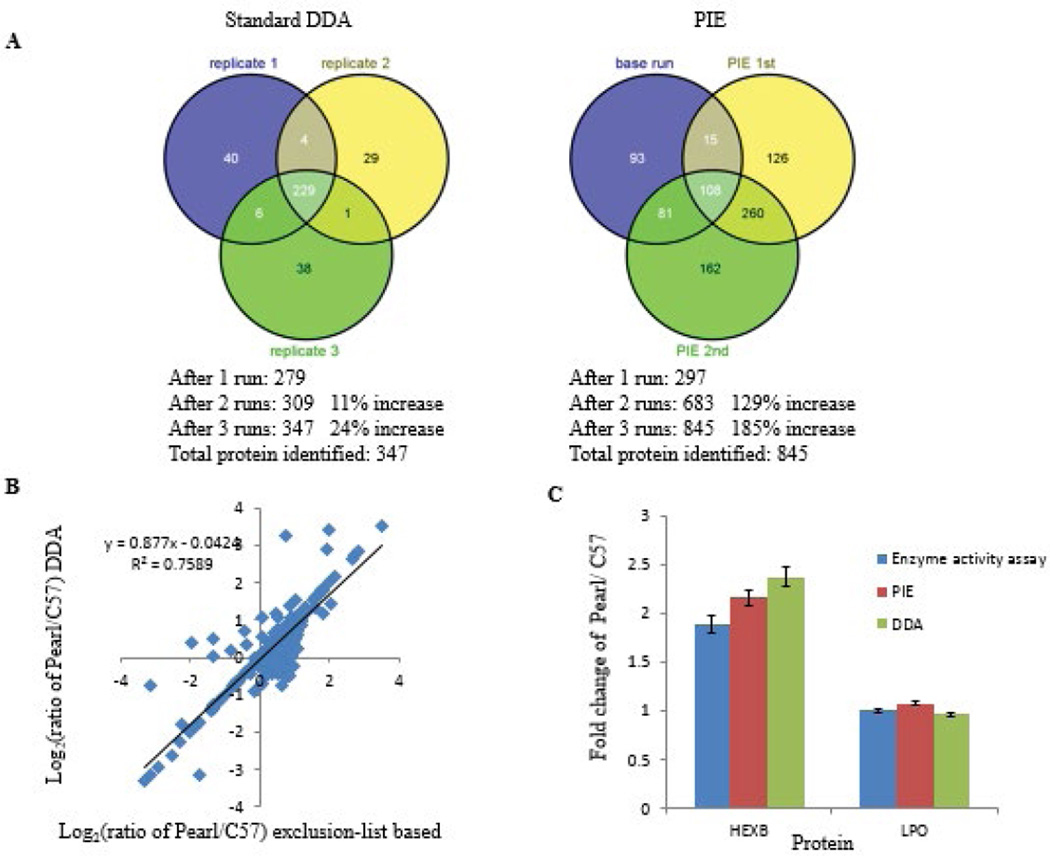
Exclusion list based-method significantly improved TMT proteome quantification compared to standard DDA method. (A) Venn diagram showing the number of proteins quantified in three LC-MS/MS analyses of tear samples using standard DDA method and exclusion list-based method. (B) Scatterplot of correlation of pearl versus C57 (Pearl/C57) protein expression ratios determined by exclusion list-based and DDA MS data acquisition methods. The common set of log2-transformed ratios of Pearl/C57 from the exclusion list-based experiment was plotted against those from the DDA experiment. (C) Quantitative profiles of HEXB and LPO using PIE, DDA and enzyme activity assay.

We identified a total of 845 proteins and 1401 peptides using the PIE workflow, while the standard DDA method only resulted in 347 proteins and 731 peptides (Figure 3A). This represents a 144% increase in the absolute number of protein identifications as a result of PIE analysis. We were able to identify a greater number of proteins and achieve a higher level of sequence coverage than that would be realized using simple replicate analyses. In common dataset, about 69% of identified proteins using PIE have increased coverage. In the PIE dataset, 45 proteins have more than 3 peptides, 62 proteins have more than 2 peptides, and 129 proteins have more than 1 peptide. In the DDA dataset, 46 proteins have more than 3 peptides, 60 proteins have more than 2 peptides, and 91 proteins have more than 1 peptide. Thus, the protein and peptide spectral-count reductions from high-abundance proteins and peptides using the PIE method led to more protein identifications overall, with less protein overlap between runs and more new peptides identified per run (Supplementary Figure 1).

Practically, PIE narrows the dynamic range of a complex proteome by excluding the most readily identified peptides, thereby improving the identification and sequence coverage of lower-abundance proteins from the next run. This strategy forces the mass spectrometer to sample new, less-abundant peptides for MS/MS analysis for the successive iterations. (Supplementary Figure 1)

We compared the average pearl/C57 protein ratios detected by the Exclusion list based study with their corresponding ratios measured by the triplicate standard DDA analyses (Figure 3B). The ratios observed by PIE and DDA were positively correlated (correlation coefficient R^2^=0.76). The slope of the regression serves as an indication of the relative sensitivity of one quantification strategy to the other. A slope of 1.0 would indicate equal sensitivity between two experiments. Using the scatter plot of log2-transformed ratio of the common proteins in the two TMT experiments, we observed good protein fold change correlations between the two methods. A slope less than 1.00 indicates that the sensitivity of PIE in the detection of relative protein expression is better than the DDA. These results indicate that improving identification comprehensiveness with PIE-based did not adversely affect the quality or confidence of peptides also easily identified in control runs.

We uploaded standard DDA and PIE dataset to Ingenuity Pathway Analysis (IPA) to evaluate the biological relevance of both datasets. The IPA uses a knowledge database derived from the literature to relate the proteins to each other based on their interaction and function. IPA could highlight the most informative and significantly overrepresented Gene Ontology terms in the dataset. Top diseases within the PIE dataset include respiratory diseases (245 proteins; p values, 9.81 × 10^−10^–2.37 × 10^−2^), immunological diseases (92 proteins; p values, 2.21 × 10^−8^–1.29 × 10^−2^), and hematological diseases (21 proteins; p values, 3.38 × 10^−8^–2.86 × 10^−2^). Top diseases within the DDA dataset include respiratory diseases (98 proteins; p values, 2.38 × 10^−6^–1.38 × 10^−2^), immunological diseases (42 proteins; p values, 2.38 × 10^−6^–1.38 × 10^−2^), and hematological diseases (18 proteins; p values, 2.38 × 10^−6^–1.38 × 10^−2^). The top biological analysis shows PIE dataset better reflects the features of HPS than standard DDA dataset.

Our further IPA analysis shows the PIE dataset could provide more specific disease-related information compared to standard DDA dataset. From the PIE dataset, 22 (reference set: 504) proteins were associated with bleeding, 41 (reference set: 1287) proteins were associated with hematopoiesis, 3 (reference set: 125) proteins were associated with pigmentation, and 8 (reference set: 174) proteins were associated with lysosomal dysfunction. However, in the standard DDA dataset only 8(reference set: 504) proteins were associated with bleeding, 14 (reference set: 1287) proteins were associated with hematopoiesis, 1 (reference set: 125) protein was associated with pigmentation, and 3 (reference set: 174) proteins were associated with lysosomal dysfunction (Supplementary Figure 2–5). From the dataset obtained with the PIE method, IPA identified biomarkers for Hermansky-Pudlak syndrome, i.e., AP3B1 and HPS3, which were undetectable in the standard DDA dataset.

IPA analyses of the data pointed to lysosomal sorting dysfunction in pearl mouse tears. We validated these findings by assaying the activities of two enzymes (β-hexosaminidase and peroxidase) in tear fluid from C57 and pearl mice (Figure 3C). The β-hexosaminidase activity in pearl mouse tears showed a significant increase (2-fold) (p<0.05) relative to C57 mouse. In contrast, no difference was observed in peroxidase activity between these 2 samples. This data is highly consistent with quantitative data of beta subunit of β-hexosaminidase (HEXB) and lactoperoxidase (LPO), which is a member of the peroxidase family secreted into tear fluid, from PIE and DDT data.

IPA analysis indicates our new method could provide better targets for future investigations on the molecular and pathological aspects of Hermansky-Pudlak syndrome. Furthermore, this method opens up the possibility to identify disease biomarkers in tear fluids from diverse animal model and clinical samples.

## Conclusion

The major aim of this study was to develop and evaluate efficient methods to gain further depth in the quantitative profiling of tear proteome, to maximize proteins quantifications while minimizing redundancies in serial analyses.

In summary, this novel, streamlined workflow overcame one of the major challenges of analyzing low abundance proteins from small samples such as tear fluid. Identifying disease biomarkers in tear fluid poses unique advantages, due to its ease of collection and the vast varieties of proteins present in the tears, making it an ideal target for biomarker discoveries. Using our streamlined PIE method, the dynamic range of analysis is improved in favor of low abundance proteins. Because our strategy relies on improved MS data acquisition, it could be used in other quantitative proteomic pipelines (label free, iTRAQ or SILAC) and other biological samples.

## Supplementary Material

Supplementary file
